# Novel compound heterozygous variants (c.971delA/c.542C > T) in *SLC1A4* causes spastic tetraplegia, thin corpus callosum, and progressive microcephaly: a case report and mutational analysis

**DOI:** 10.3389/fped.2023.1183574

**Published:** 2023-07-12

**Authors:** Feda E. Mohamed, Mohammad A. Ghattas, Taleb M. Almansoori, Mohammed Tabouni, Ibrahim Baydoun, Praseetha Kizhakkedath, Anne John, Hiba Alblooshi, Qudsia Shaukat, Fatma Al-Jasmi

**Affiliations:** ^1^ Department of Genetics and Genomics, College of Medicine and Health Sciences, United Arab Emirates University, Al Ain, United Arab Emirates; ^2^ College of Pharmacy, Al Ain University, Al Ain, United Arab Emirates; ^3^ AAU Health and Biomedical Research Center, Al Ain University, Abu Dhabi, United Arab Emirates; ^4^ Department of Radiology, College of Medicine and Health Sciences, United Arab Emirates University, Al Ain, United Arab Emirates; ^5^ Department of Pediatrics, Tawam Hospital, Al Ain, United Arab Emirates

**Keywords:** spastic tetraplegia, microcephaly, amino acid transporter, spasticity, seizure

## Abstract

Spastic tetraplegia, thin corpus callosum, and progressive microcephaly (SPATCCM) are linked to *SLC1A4* genetic variants since the first reported case in 2015. *SLC1A4* encodes for the neutral amino acid transporter ASCT1 which is involved in the transportation of serine between astrocytes and neurons. Although most of the reported cases are of Ashkenazi Jewish ancestry, SPATCCM has also been reported in Irish, Italian, Czech, Palestinian, and Pakistani ethnicities. Herein, we report two Pakistani male siblings from a non-consanguineous marriage presented with global developmental delay associated with spastic quadriplegia, microcephaly, and infantile spasm. Since infancy, both siblings suffered from microcephaly with brain MRI demonstrating generalized atrophy of the frontal, temporal, and parietal lobes with a prominence of the subarachnoid spaces, widening of the Sylvian fissures, and enlargement of the ventricular system not compatible with the chronological age of both patients associated with thinning of the corpus callosum. Whole-exome sequencing of both affected brothers revealed novel compound heterozygous variants in the *SLC1A4* gene (NM_003038) segregating from their parents. The maternal c.971delA (p.N324Tfs*29) deletion variant disturbs the transcript reading frame leading to the generation of a premature stop codon and its subsequent degradation by nonsense-mediated mRNA decay as detected through expression analysis. The paternal c.542C > T (p.S181F) missense variant was predicted deleterious via multiple in silico prediction tools as the amino acid substitution is speculated to affect the overall ASCT1 structural confirmation due to the loss of an H-bond at the core of the protein at this position which might affect its function as concluded from the simulation analysis. The presented cases expand the genetic and clinical spectrum of ASCT1 deficiency and support the importance of including *SLC1A4* gene screening in infants with unexplained global neurodevelopmental delay regardless of ethnicity.

## 1. Introduction

Alanine Serine Cysteine Transporter 1 (ASCT1; P43007-1) is a neutral amino acid transporter protein that is mainly responsible for the transport of l-Serine amino acids from astrocytes to neuronal cells in a Na^+^-dependent mechanism (1). L-Serine is a nonessential amino acid that can be provided through diet and protein degradation. Because of its poor permeability through the blood-brain barrier, l-Serine is synthesized in the brain by astrocytes and shuttled to neurons through ASCT1, serving as a precursor for l-cysteine, phosphatidyl-l-serine, sphingolipids, and different neuromodulators (2). Therefore, ASCT1 is crucial for the early development and function of the neuronal system and is ubiquitously expressed in the brain (3, 4). The transporter protein is expressed by the solute carrier family 1 member 4 (*SLC1A4*) gene (HGNC:10942; NM_003038.5) mapped to chromosome 2p14 (5).

Genetic defects in the *SLC1A4* gene (MIM: 600229) have been linked to an autosomal recessive neurodevelopmental disorder (MIM: 616657) that is mainly characterized by early infantile onset of spastic tetraplegia, thin corpus callosum, and progressive microcephaly (SPATCCM) leading to severe and progressive early developmental delay (6). The first SPATCCM case was published in 2015 reporting two siblings from a consanguineous Ashkenazi Jewish family carrying the founder p.E256K mutation that has a carrier frequency of 0.7% in the population (7). Later, multiple other disease-causing variants like p.L315fs and p.R457W were detected in eight or more families of the same origin, strongly suggesting including the *SLC1A4* gene in the carrier screening panel of this community. However, the phenotype has been reported in families of European and Middle Eastern origins, supporting the fact of investigating for ASCT1 deficiency in cases with unexplained neurodevelopmental disorders regardless of ethnicity (8, 9). Collectively, there are a total of 10 *SLC1A4* genetic variants reported in the Human Gene Mutation database (HGMD) that are linked to the autosomal recessive SPATCCM phenotype of which are three nonsense, four missense, two deletion, and one insertion variants (10).

So far, there is only one case of SPATCCM reported in the Pakistani population that is associated with seizure disorder due to a nonsense variant in the *SLC1A4* gene (c.573T > G; p.Y191*) (8). Herein, we are reporting the second case of novel compound heterozygous *SLC1A4* mutations identified by whole-exome sequencing (WES) in two Pakistani siblings of a non-consanguineous family. The pathogenicity of the detected variants was confirmed through multiple in silico molecular dynamics (MD) simulations and expression analyses.

## 2. Materials and methods

### 2.1. Ethical consideration

This study was approved by the Abu Dhabi Health Research and Technology Committee, reference number DOH/CVDC/2021/1318 as per national regulations. Affected patients were identified by the neurology and metabolic team at Tawam Hospital, Abu Dhabi, for clinical evaluation and follow-up. All participants signed an informed consent form to participate in this study.

### 2.2. Whole-Exome sequencing (WES)

Genomic DNA (gDNA) from whole blood (collected in EDTA tubes) was extracted using the QIAcube automated DNA extraction system with the QIAmp DNA Mini kit (Qiagen). The quantity and quality of genomic DNA were assessed by both spectrophotometric (Nanodrop, Thermo Fisher Scientific, Waltham, MA, USA) and fluorometric methods (Qubit 4.0 fluorometer, Thermo Fisher Scientific, Waltham, MA, USA). Genomic DNA was fragmented by a Covaris LE-220 plus ultrasonication system (Covaris Inc., Woburn, MA, USA). Whole-exome sequencing library preparation and target enrichment were performed using TruSeq DNA Exome (Illumina Inc., San Diego, CA, USA.) kit according to the manufacturer's protocol. The libraries were sequenced with paired-end reads (2 × 150 bp) on the NovaSeq6000 system (Illumina Inc., San Diego, CA, USA). Read mapping (GRCh37/hg19 reference assembly), alignment, and variant calling were performed using a combination of in-house developed pipelines and the Illumina DRAGEN Bio-IT platform. Variant annotation and filtration were performed using the VarSeq software (Golden Helix Inc, Boseman, MT) using a customized pipeline. The following databases and *in silico* algorithms were used to annotate and evaluate the impact of the variant in the context of human disease: gnomAD, ClinVar, HGMD, OMIM, dbSNP, NCIB RefSeq Genes, the Exome Aggregation Consortium (ExAC) Gene Constraints, Sorting Intolerant From Tolerant (SIFT), PolyPhen2, PhyloP, GERP++, GeneSplicer, MaxEntScan, NNSplice, and CADD scores. All disease-causing variants in ClinVar [http://ncbi.nlm.nih.gov/clinvar], Human Genome Mutation Database [HGMD; http://hgmd.cg.ac.uk] (10), as well as all variants with minor allele frequency (MAF) of less than 1% in the gnomAD database (11) in the coding regions and exon/intron boundaries ± 20 bp in the target genes, were considered. For the clinical correlation of the identified variants, relevant inheritance patterns based on clinical information and family history provided by the referring physician were used. The variants were interpreted according to the criteria specified by ACMG guidelines (12) and patient phenotype.

### 2.3. PCR amplification and validation by sanger sequencing

*SLC1A4* gene variants detected via WES were confirmed via Sanger sequencing. Primers for exon 2; NM_003038.5: c.542C > T (forward; 5′- AGTTCAAGGCTGCAGTGAGCTG-3′ and reverse; 5′- ACCATCTCTCCCTGTTCTACC-3′) and exon 5; NM_003038.5:c.971delA (forward; 5′- AGGAAGGACCTGCATCTCTCAC-3′ and reverse; 5′-CACCTGGTTCCATGTTAACATG-3′) were designed via Primer3Plus (http://www.bioinformatics.nl/cgi-bin/primer3plus/primer3plus.cgi/) and purchased from Metabion Inc. (Germany). PCR amplification and Sanger Sequencing were carried out as previously performed (13).

### 2.4. In silico analysis and the impact of the variants on protein structure and function

The *SLC1A4* gene transcript (ENST00000234256) and protein sequence (NM_003038; P43007) were obtained from the Ensembl and UniProt databases, respectively. The ExPASy translate tool was used to predict the impact of the filtered variants from WES on the protein sequence. Various in silico prediction tools were employed to assess the effect of the detected genetic variants on protein function. The SIFT algorithm determined whether the variants affected ASCT1 protein function based on a score threshold of 0.05 (14). The PolyPhen-2 algorithm evaluated the impact on ASCT1 structure and function, with scores categorized as benign (0–0.15), possibly damaging (0.15–0.9), or confidently damaging (0.9–1) (15). The PROVEAN software assessed the influence of ASCT1 amino acid substitution on biological function, with a score below −2.5 indicating a deleterious variant (16). Lastly, the MutationTaster online tool was utilized to evaluate whether these variants could be disease-causing (17).

### 2.5. Real-time PCR

Total RNA was isolated from EDTA-collected whole blood samples using TRIzol Reagent (Life Technologies). Total RNA was precipitated, purified, and eluted using a QlAamp RNA blood mini kit (Qiagen). Total RNA was subjected to reverse transcription using Promega GoScript reverse transcriptase kit by following the manufacturer's instructions. Quantitative real-time PCR was performed on a QuantStudio 7 Flex (Applied Biosystems) real-time PCR machine. TaqMan real-time PCR assays (Life Technologies) for SLC1A4 (Hs00983079_m1) were used for analyzing ASCT1 expression and as an internal reference control, GAPDH (Hs02758991_g1) was used, according to the manufacturer's protocol. Gene expression was analyzed by relative quantification, normalized to GAPDH (reference gene) expression, and calculated using the comparative Ct (ΔCt) method via the QuantStudio Real-Time PCR software v1.2. The relative expression presented in the bar graph is a representation of the mean of three independent reaction runs, where error bars indicate S.E.M.

### 2.6. Molecular dynamics simulation

#### 2.6.1. Stability Study

The ASCT1 crystal structure was obtained from the protein data bank (PDB ID: 7P4I2) (18). The ASCT1 structure was checked for missing residues and atoms, then it was protonated via the protein preparation module in the MOE software package (19). Consequently, the ASCT1 structure was mutated from serine to phenylalanine at position 181 by the Residue Scan module in MOE, which then produced the 3D structure of the mutant form (19).

#### 2.6.2. Molecular dynamics protocol

The wild-type and mutated solvated systems of the ASCT1 transporter were built separately via XLeap in the AmberTools program (20). Both systems were energy-minimized and simulated (via molecular dynamics) via pmemd in the Amber18 software package (20). Minimization was conducted via two consecutive steps of 1,000 cycles each by applying 2 kcal/mol restraint forces on the protein atoms only in the first stage. The resultant systems were heated over the time course of 20 ps from 0 to 300 K under NVT conditions using a Langevin thermostat and 2 kcal/mol restraint force (on protein atoms). Subsequently, the solvated systems' densities were equilibrated for 100 ps and used again at 2 kcal/mol to restrain the forces on protein atoms. MD simulations were conducted for the course of 100 ns using an average pressure of 1 atm under NPT conditions, with a relaxation time of 2 ps. The average temperature was set to 300 K, and it was controlled by a Langevin thermostat (using a collision frequency of 1.0 ps^−1^). The Particle mesh Ewald algorithm was employed to run all explicit solvent calculations, with a cutoff of 10 Å for long-range electrostatics (21). The SHAKE algorithm Hydrogen was used to constrain hydrogen atoms’ covalent bonds during all MD simulations. Clustering was done to identify the top clustered conformation using the CCPTRAJ module, where the Epsilon value was set to 1.5 after all frames were stripped out of solvent molecules and ions (22).

### 2.7. Data availability statement

Variant data have been submitted to ClinVar with the following submission IDs: SUB11885160 for the deletion (c.971delA) variant and SUB11885207 for the missense (c.542C > T) variant. The data that support the findings of this study are available upon request from the corresponding author.

## 3. Clinical presentation

**Figure 1:**
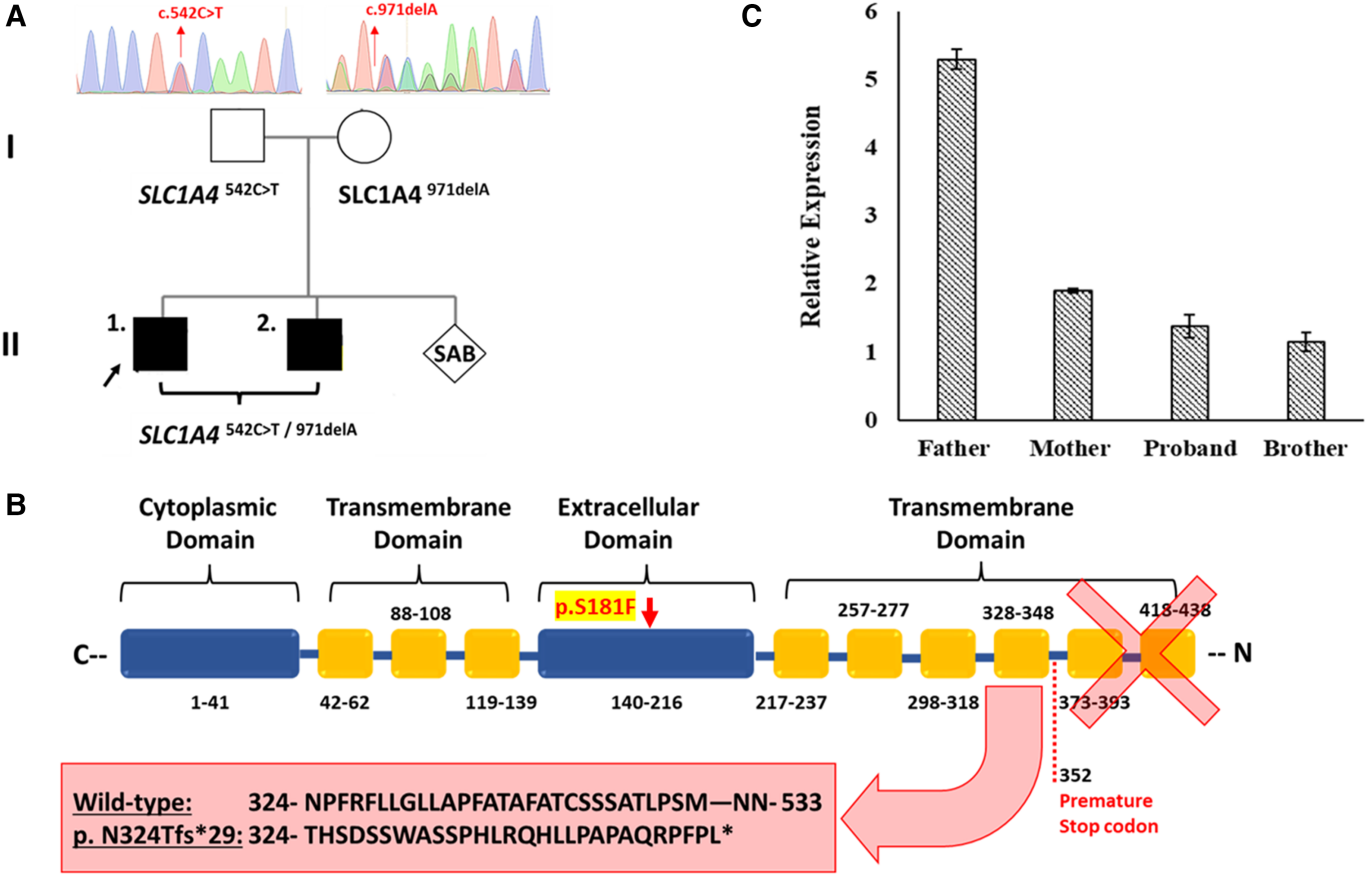
The effect of the detected *SLC1A4* genetic variants on ASCT1 expression. (**A**) Genetic pedigree chart of the affected family showing the reported variants inheritance pattern. (**B**) Schematic illustration of ASCT1 protein showing the position at which the premature termination is generated due to c.971delA deletion variant and its subsequent loss of three functionally critical transmembrane domains. The position of the p.S181F amino acid substitution in the extracellular domain of ASCT1 is highlighted in yellow. (**C**) Relative expression of the *SLC1A4* gene in blood samples of all family members measured *via* RT-PCR confirming the pathogenicity of both reported variants. Error bars represent ± SEM of three independent experiments.

In this study, two male siblings of Pakistani origin were presented at the metabolic clinic with a neurodevelopmental disorder characterized by severe global developmental delay since birth. They were diagnosed with SPATCCM through clinical examination and molecular analyses. The family had no previous history of the disease. Parents are non-consanguineous and had one miscarriage during early pregnancy for unknown reasons (Figure 1A). The diagnosis in both siblings was confirmed via whole-exome sequencing (WES) at UAEU genomic lab that detected novel compound heterozygous variants in the *SLC1A4* gene. The reported variants were detected in both affected siblings segregating from their parents as confirmed by Sanger Sequencing. The pathogenicity of the novel variants was further confirmed via functional and structural analyses as described in detail below.

### 3.1. Patient 1 (proband)

6-year-old Pakistani boy born to non-consanguineous parents presented with an infantile-onset global developmental delay, spastic quadriplegic, seizures, acquired microcephaly, and failure to thrive. Metabolic workup and chromosomal analyses (karyotype and chromosomal microarray analysis) were unremarkable. Compound heterozygous variants (deletion and missense) in *SLC1A4* were detected by WES, which was confirmed by Sanger Sequencing.

The proband was born at term with an unremarkable perinatal history and 3 kg weight. He smiled at 3 months, rolled over at 10 months, and was able to sit at 11 months, but he could not sit after his first febrile seizure. His seizures were noticed during a febrile illness at 16 months of age for which he was managed with levetiracetam. He started to regain multiple abilities like rolling over and started sitting again with a curved back at 18 months of age. He could not crawl or pull himself to a standing position or cruise around. He was able to reach for objects, hold, and transfer them from hand to hand, but he did not have a pincer grasp. His speech skills were delayed, and he could not speak but communicate through screaming and sounds. He had good eye contact but was socially anxious toward strangers. He was not interacting with others but was able to clap and wave bye. His development was affected in all aspects. He was managed with baclofen and routine botulinum toxin injections for his muscle spasticity where the lower limbs were severely affected. The spasticity was progressive; it started in the lower limbs and progressed to the upper limbs as well. Botox injection improved the muscle tone, but continuous administration was required to maintain the improvement. His condition is severely progressive, keeping him wheelchair dependent. The proband was seizure-free on Levetiracetam until 5 years of age when he started new types of seizures. In addition to myoclonic seizures, the proband's mother reported seizure episodes that resembled tonic spasms. These episodes were progressive, reaching up to 8 to 10 tonic seizures during his sleep.

The proband had a failure to thrive and had recurrent aspiration pneumonia because of feeding difficulties which were confirmed by swallowing studies. He is currently fed by a nasogastric (NG) tube. He had a ventricular septal defect (VSD) and a history of recurrent urinary tract infection (UTI). He was admitted to the hospital for acute gastroenteritis with prerenal azotemia which was managed with intravenous fluid and supportive management.

The patient's (6 years) current physical parameters are a weight of 15.8 kg <3 percentile and height of 101 cm <3 percentile. He is wheelchair-bound and has microcephaly (head circumference: 46 cm), normal heart rate, regular rhythm, and normal peripheral perfusion with no reported edema or gallop. A grade III systolic murmur was detected in the left sternal border. He has significant spasticity in all four limbs; the tone is on a modified Ashworth scale of 1+ in the upper limbs and three in the lower limbs (the lower limbs are more severely affected). Deep tendon reflexes are exaggerated in all four limbs and bilateral ankle clonus is present. He has scissoring of his legs. Cranial nerves are grossly intact. There was no nystagmus and no intentional tremors.

**Figure 2:**
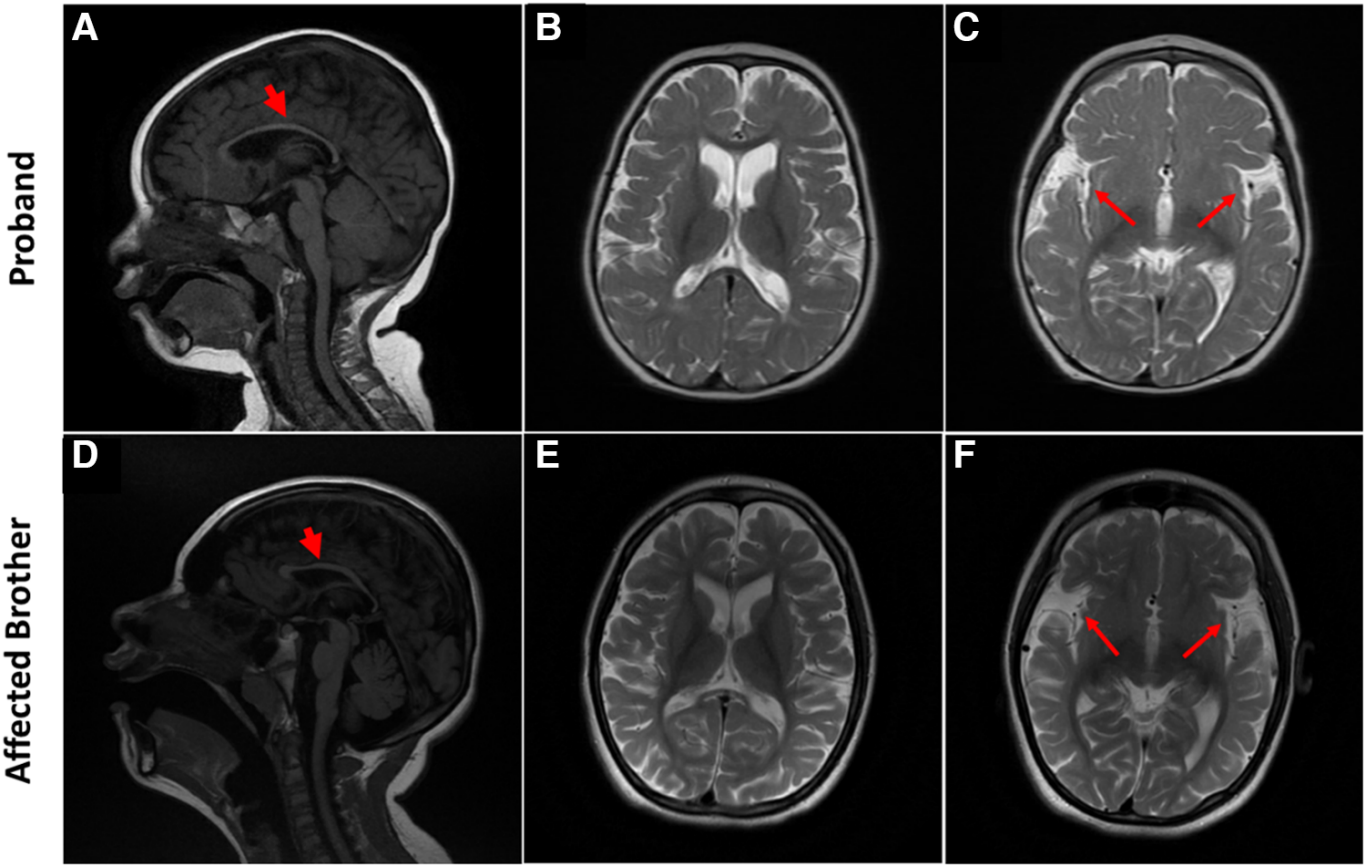
Brain MRI characteristic of SPATCCM in the proband (top panel) and his affected brother (lower panel). (**A**) Sagittal T1 weighted image demonstrates significant thinning of the corpus callosum. (**B**) Axial T2-Weighted Image demonstrating atrophy of the frontal and temporal lobes with a prominence of the subarachnoid spaces. (**C**) Widening of the Sylvian fissures. (**D**) Sagittal T1-weighted image demonstrates significant thinning of the corpus callosum and mild atrophy of the cerebellum. (**E**) Axial T2-Weighted Image demonstrating generalized atrophy of the frontal, temporal, and parietal lobes with a prominence of the subarachnoid spaces; enlargement of the ventricular system is not compatible with the chronological age of the patient. (**F**) Widening of the Sylvian fissures.

At 5 years of age, the proband's electroencephalogram (EEG) was performed, showing abnormal patterns. The awake record showed a slow background consisting of mixed delta and theta activity (mostly delta). During drowsiness and while sleeping, the EEG showed a frequent high amplitude of generalized and sharp spikes/poly-spikes, and slow waves were noted, occurring at ≲2 Hz/sec. At times, these epileptiform discharges occur in runs. The photic paroxysmal response was noted at certain frequencies with spikes and slow wave discharges predominantly in the posterior region. One episode of myoclonic jerk was noted during sleep, coinciding with spikes/poly-spikes and slow waves in EEG. Another episode of right upper limb movement (jerk) was noted during a long run of epileptiform discharges. Tonic seizures were not noted at the time of EEG recording. EEG was in favor of diffuse cerebral dysfunction and encephalopathy and suggestive of Lennox Gastaut syndrome. Brain MRI showed generalized atrophy of the frontal, temporal, and parietal lobes with a prominence of the subarachnoid spaces, widening of the Sylvian fissures, and enlargement of the ventricular system with thinning of the corpus callosum and mild atrophy of the cerebellum which were not compatible with the chronological age of the patient (Figures 2A–C). The gray-white matter differentiation was seen to be preserved. Unfortunately, no follow-up imaging was done at an older age for reanalysis.

Seizures were managed by adding sodium valproate to his medication  since he was 5 years old and currently, he is seizure free on the following antiepileptic medications: Clobazam (which was started at the age of 4 years) 2.5 mg BID (0.25 mg/kg/day) and Sodium Valproate 300 mg BID (30 mg/kg/day). Levetiracetam was slowly tapered off. His sleep interruptions were managed with 9 mg of melatonin. Symptoms were slightly improved with serine supplement as per the mother's report, but clinically, no improvement was noted.

### 3.2. Patient 2 (proband's older brother)

A 14-year-old boy presented with profound global developmental delay, sleep disturbances, and severe spasticity in both lower limbs resulting in wheelchair dependence. The long clinical diagnostic journey revealed compound heterozygous genetic variants (deletion and missense variant) in the *SLC1A4* gene inherited from his parents as revealed by WES.

The perinatal history was unremarkable; the mother had a normal pregnancy and gave birth by normal vaginal delivery without any postnatal problems and required no resuscitation at birth. He had a history of infantile spasms and seizures since the age of 7 months, which persisted up to the age of 3–4 years and then stopped at which his parents discontinued his antiepileptic medications. The seizure was generalized with an episodic activity that usually lasts for a few seconds. It used to occur as multiple episodes mostly upon waking up from sleep. He had a history of feeding difficulties and recurrent upper respiratory tract infections. The patient was reported with repetitive habitual movements of his legs. He experienced significant developmental delays since birth. Although he started sitting at 6 months, he never crawled or walked. He was unable to stand even with support, and he is wheelchair-bound. He was and still is unable to perform any useful activities of daily living due to severe cognitive defects and impaired fine motor skills. He can reach objects and transfer them from one hand to another but has no pincer grasp. His receptive and expressive language was not developed due to severe cognitive delay; he never formed words or simple sentences but only produces sounds and cannot follow simple commands. His vision is normal; he can fix his gaze and visually follow in all directions.

He is normally alert and hyperactive, communicating with incomprehensible sounds and screaming at a time without obvious reasons. He has microcephaly (46 cm head circumference at the age of 14) but no dysmorphic features. His weight is 27 kg, *Z*-Score is −4.85; height is 142 cm, *Z* score is −3.05. He has a normal heart rate, regular rhythm, and no cardiac murmur. Respiratory and abdomen examination is within normal limits. An examination of the spine revealed thoracolumbar scoliosis. He has reduced muscle bulk, increased tone in both upper limbs (on MAS 2) and severe spasticity with bilateral muscle contractures at both knees and ankles (Tone on MAS 4). Deep tendon reflexes are exaggerated in all four limbs, and bilateral ankle clonus is present. Cranial nerves are grossly intact and there are no cerebellar signs (no nystagmus, no ataxia, and no intentional tremors). Like his sibling but to a lesser extent, Brain MRI showed atrophy of the frontal and temporal lobes with a prominence of the subarachnoid spaces and widening of the Sylvian fissures bilaterally associated with thinning of the corpus callosum (Figures 2D–F). The grey-white matter differentiation is preserved.

Furthermore, the patient's initial EEG showed abnormal consistency with an interictal record of modified hypsarrhythmia. The background consisted of delta activity disorganized at places. There were bursts of high amplitude and slow waves seen at periodic intervals on both sides lasting for 1–2 s at places. These bursts were followed by voltage suppression. Random spikes and slow waves were also noticed. EEG was repeated at 7 years of age during his sleep, showing mixed theta and delta activity. Occasional episodes of focal sharp wave activity were seen bilaterally. These findings proved and supported the diagnosis of a convulsive tendency.

Basic metabolic workup including ammonia, lactate, homocysteine, urine organic acid, amino acid, and acylcarnitine was normal as well as chromosomal microarray analysis. The patient was managed with baclofen and botox injections regularly for the management of muscle spasticity. Currently, he is seizure-free and not on any antiepileptic medications.

## 4. Results and discussion

### 4.1. Whole-exome sequencing identified novel compound heterozygous variants (c.971delA and c.542c > T) in the *SLC1A4* gene of both affected patients

We have performed WES for both affected siblings and their parents revealing compound heterozygous variants with a deletion and missense variant in both patients (Figure 1A). Both detected variants were neither reported in gnomAD nor 1,000G databases (23, 24). The c.971delA deletion lies within exon 5 (chr2:65243744_65243744delA) and is segregated from the mother as confirmed by Sanger Sequencing. The mutation is predicted to result in the degradation of the mRNA transcript through the non-mediated decay (NMD) machinery as indicated by the Mutation Taster tool (17). However, if it managed to evade NMD, the mutated ASCT1 harboring a deletion of a single nucleotide at this position will disturb the reading frame leading to a frameshift mutation and the generation of a premature stop codon at position 1,056 instead of 1,599 in the wild-type transcript (Table 1). At the protein level, the frameshift will start at position 324, substituting the wild-type Asn with Thr disturbing the rest of the amino acids (AAs) sequence leading to the generation of a premature stop codon after 29 residues (p.N324Tfs*29). The premature termination will result in the loss of 181 AAs (Figure 1B). Overall, this genetic change will lead to the loss of three (out of a total of six) N-terminal transmembrane domains, five serine phosphorylation sites (at positions: 502, 507, 521, 527, and 530), and five lysine ubiquitination sites (at positions: 483, 484, 493, 501, and 528) (3, 25). ASCT1 has three sodium binding sites (site 1—E465 and D467; site 2—T376 and V418; site 3—F121, T124, T125, N378, and D380) that will most probably be lost or affected due to the underlying damage (3). More importantly, residues of the binding sites (C343, S345, D452, D456, T459, T460, and N463) will also be lost due to the underlying premature termination (25). Multiple other *SLC1A4* premature termination mutations have been reported previously with similar effects resulting in non-mediated decay like p.Y191*, p.W453*, and p.L315Hfs*42 noting that p.N324Tfs*29 variant identified in this case study is located upstream to most of the previously reported pathogenic mutations (7–9).

**Table 1:** In silico prediction analysis of c.971delA variant effect on ASCT1 function and structure.

		c.971delA (p.N324Tfs*29)
Exon		5
gnomAD and/or 1,000G		Not reported
Mutation taster		NMD due to frameshift and generation of a premature stop codon
Conservation		Conserved
Stop codon position (WT/Mut)	CDS (Coding Sequences)	1,599/1,056
	Protein	533/352
ExPASy translate		Loss of 181 amino acids

The paternal c.542C > T missense variant, on the other hand, lies within exon 2 of *SLC1A4* and results in the substitution of the wild-type serine (Ser) to phenylalanine (Phe) at position 181 at the protein level. The amino acid change at this position is predicted to be damaging and disease-causing by multiple *in silico* prediction tools (Table 2). It is speculated to affect the protein folding structure and conformation as Ser181 is part of the extracellular domain of the transporter protein that contains critical residues vital to its function (3). The wild-type residue is buried in the core of the ASCT1 protein and located within a loop that links two alpha helixes; however, the mutant amino acid is bulkier than the wild-type and most probably will not fit in this position, disturbing the structure as speculated by the HOPE project tool (26).

**Table 2:** In silico prediction analysis of c.542C > T variant effect on ASCT1 function and structure.

		c.542C > T (p.S181F)
Exon		2
gnomAD and/or 1,000G		Not reported
Mutation taster		Disease-causing due to amino acid change sequence
SIFT*	Effect	Affect protein function
	Score	0.04
Conservation		Highly conserved
PROVEAN**	Effect	Deleterious
	Score	−3.904
HOPE		Predicted to disturb correct folding due to the loss of H-bond in the core of the protein
PolyPhen2^†^	Effect	Probably damaging
	Score	0.988

* The score ranges from 0 to 1. The amino acid substitution is predicted damaging if the score is ≤0.05, and tolerated if the score is >0.05.

** Scores ≤−2.5 are considered “deleterious”. Scores  > −2.5 are considered “neutral”.

^†^ The score ranges from 0.0 (tolerated)—1.0 (deleterious).

### 4.2. p.N324Tfs*29 variant leads to non-mediated decay and loss of ASCT1 expression

To further prove the predicted effect of the detected deletion, ASCT1 expression has been assessed in all family members. Extracted mRNA samples from whole blood have been collected and transformed into cDNA to measure the relative expression of the *SLC1A4* gene in the affected patients and their parents via quantitative real-time PCR (RT-PCR). The TaqMan probe used for the intended purpose was chosen to bind ahead (at exon 2) to the deletion mutation position (exon 5), making sure to detect the transcript if it was not directed for degradation. Since the mother was a heterozygous carrier for the c.971delA variant, *SLC1A4* relative expression was around three folds less than the father who is a heterozygous carrier for the substitution variant (Figure 1C). As a result, the expression analysis confirmed the damaging effect of the deletion mutation leading to its degradation via NMD. Like the mother, both patients showed relatively similar *SLC1A4* expression patterns (Figure 1C). Interestingly, the detected expression amount detected in the mother by the wild-type allele was enough to compensate for the observed loss; hence, she was clinically normal. However, the amount expressed by the *SLC1A4*^S181F^ allele in both patients did not recompense for the decreased expression.

### 4.3. The predicted loss of function in the p.S181f mutated ASCT1 is due to an overall structural and conformational distortion

**Figure 3:**
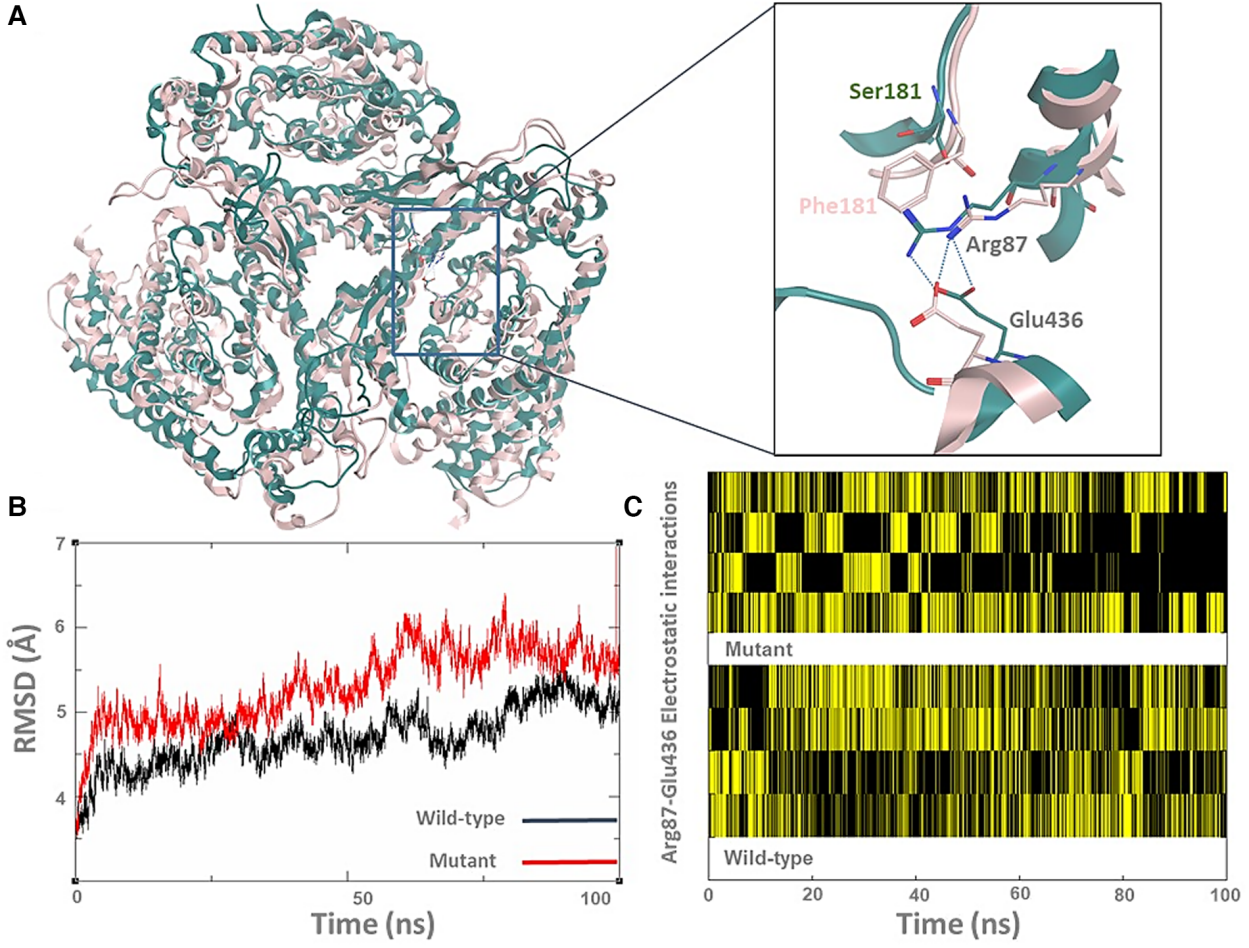
The p.S181F missense variant detected that the loss-of-function effect is due to a structural conformational defect. (**A**) The top-cluster conformations of the wild type (green cartoon) and mutated structures (pink cartoon) of the ASCT1 transporter were obtained from 100 ns MD simulations. The zoom-in picture shows the S181F mutation along with a nearby interrupted interaction between Arg87 and Glu436 (hydrogen bonding as a blue dotted line). (**B**) The backbone RMSD analysis of both ASCT1 transporter. (**C**) The Arg87-Glu436 electrostatic interaction pattern of the ASCT1 wild-type (bottom) compared to the mutant form (top) over the course of the MD simulation.

Fortunately, the ASCT1 crystal structure has recently been revealed, which allowed us to study the effect of the p.S181F mutation on the transporter quaternary structure (3). As shown in Figure 3A, the top clustered conformations of the wild-type and mutant forms were sampled and aligned on top of each other; then backbone RMSD (Root mean square deviation) (Figure 3B) and hydrogen bonding analyses (Figure 3C) were conducted to view the effect of the p.S181F mutation on the overall structure and certain electrostatic interaction patterns within the nearby residues.

Although the Ser181 side chain appears not to be involved in solid interactions with the neighbor amino acids, replacing it with a bulkier residue such as phenylalanine seems to heavily disturb the arrangement of the surrounding secondary structure of the transporter (Figure 3A). This indeed was influenced by disrupting the internal network of the transporter via losing or gaining key interactions that are generally responsible for preserving the tertiary structure of the protein. For instance, the observed hydrogen bonding between Arg87 and Glu436 was disturbed by the large size of the Phe181 side chain in the mutant form, as shown in Figure 1A. The quantitative analysis of the Arg87 and Glu436 interactions over the course of the MD simulations shows that four electrostatic interactions with various intensity are usually taking place in the wild-type form (Figure 1C). Interestingly, these electrostatics seem to be disrupted in the mutant form, particularly in the second half of the MD simulations where the occurrence of two interactions is still evident while the occurrence of the other two interactions seems to be minimal and disruptive. The overall impact of the mutation on the transporter structure is also evident by the RMSD analysis of the backbone structure of both forms (Figure 1B), where the mutant form shows higher RMSD values (>5 Å) than that in the wild type which preserved it to less than 5 Å in most of the simulations. To sum up, the MD simulations showed that the S181F mutation damaged the overall protein structure and, possibly, the transportation function of ASCT1. Nonetheless, *in-vitro* assays are still needed to establish the correlations shown between the clinical data and the molecular modeling findings described in this study.

Several previously reported ASCT1 amino acid substitutions have shown similar structural defects as reported in our study (3). The simulation analysis of the p.E256K variant predicted the *de novo* formation of a salt bridge between the mutated Lys256 and Glu243 located in the fifth transmembrane domain disturbing the overall conformation of the protein. The obtained simulation analysis came in line with the reduced substrate uptake (30%–40%) performed for the same mutant through *in vitro* characterization in HEK293 cells (7). The p.R457W mutant, on the other hand, showed a complete loss of substrate uptake due to the larger sidechain of the mutated amino acid that leads to the distortion of the transporter substrate binding site as a result of its interaction with Cys343 (3, 7). The substrate binding pocket of p.G381R-mutated ASCT1 was markedly altered due to the destabilization and rearrangement of nearby critical domains (3). The wild-type glycine at this position is part of a highly conserved motif that is involved in the formation of sodium binding sites and plays a role in the coupling of sodium and substrate binding.

## 5. Conclusion

This study reported the second and third *SLC1A4* mutations in the Pakistani population. ASCT1 deficiency is not limited to the Ashkenazi Jewish population and it should be considered regardless of ethnicity in cases with an unexplained neurodevelopmental disorder associated with similar clinical implications (8). ASCT1 knock-out mice have been created and well-analyzed systematically and phenotypically by Kaplan et al.'s group in 2018 (27). The availability of such a model that mimics patients with mutations in the *SLC1A4* gene will facilitate a better understanding of the disease's clinical complications and may aid the development of therapeutic interventions to prevent or slow down the prognosis of the neurodegenerative effects of the underlying disease.

## Data Availability

The datasets presented in this study can be found in online repositories. Variant data have been submitted to ClinVar with the following submission IDs: SUB11885160 for the deletion (c.971delA) variant and SUB11885207 for the missense (c.542C>T) variant. The data that support the findings of this study are available on request from the corresponding author.
